# Online Health Behavior and Disease Management Programs: Are We Ready for Them? Are They Ready for Us?

**DOI:** 10.2196/jmir.7.3.e27

**Published:** 2005-07-01

**Authors:** Kerry E Evers, Carol O Cummins, James O Prochaska, Janice M Prochaska

**Affiliations:** ^2^Cancer Prevention Research CenterUniversity of Rhode IslandKingston, RIUSA; ^1^Pro-Change Behavior Systems, IncWest Kingston, RIUSA

**Keywords:** Health behavior, Internet, disease management, health promotion, evaluation studies

## Abstract

Advancing the science and practice of health promotion and disease management on the Internet requires a systematic program of research examining the population impact of such programs. With impact described as the combination of effectiveness and participation, such research needs to include the examination of the quality and effectiveness of programs that are available to the general public, as well as descriptive and predictive knowledge about population readiness to participate in such programs.

There have been few studies examining the quality of interactive health behavior change (HBC) programs on the Internet, and even fewer investigations of the effectiveness of such programs. Based on the review of over 300 HBC programs on the Internet using the “5 A's” of Health Behavior Change on the Internet (HBC-I Screener), which represent standard minimum guidelines for evaluation, it appears HBC on the Internet is in the early stages of development. As health behavior change on the Internet matures from the provision of health information to meeting the requirements necessary to produce health behavior change, and as program developers take advantage of the interactive nature of the Internet, the basic screening and expanded evaluation criteria developed in this project will provide templates for both consumers and developers of programs. The second component necessary for evaluating the impact of HBC on the Internet is the extent to which the population is ready to participate in such programs. We need to move beyond a narrow focus on early adopters and produce a population perspective that includes those not ready, those getting ready, and those ready to use such programs, as well as those already participating. By understanding participation levels of such programs, and what drives this participation, the development and dissemination of practical tailored and targeted interventions can help maximize population participation in Internet programs for health behavior change.

## Introduction

Advancing the science and practice of health promotion and disease management on the Internet requires a systematic program of research examining the population impact of such programs. With impact described as the combination of effectiveness and participation, such research needs to include the examination of the quality and effectiveness of programs that are available to the general public, as well as descriptive and predictive knowledge about the population readiness to participate in such programs. This paper describes initial research examining the two areas that affect the impact of Internet based programs: (1) the status of health behavior change on the Internet, including the types and quality of sites available; and (2) individuals' readiness for using the Internet for health behavior change.

## Are We Ready for Them?

Although several guidelines for evaluating health on the Internet have been published (for a sample list see [1]), few of those include specific criteria relevant to the area of health behavior change and disease management. Established criteria have often been designed specifically for websites that provided health information rather than programs aimed at helping individuals manage their health. As part of a larger study examining the impact of health behavior change on the Internet, a set of screening criteria was adapted from the Public Health Service's *Clinical Practice Guideline for Treating Tobacco Use and Dependence* [2]. The “5 A's” portion of those guidelines represent five major, but brief, intervention steps that can be used in the primary care setting for those patients who use tobacco. The “5 A's” represent generic counseling steps that can be used for most health behavior risks and that form the basis for the development of brief criteria for the basic elements needed in Internet programs designed for health behavior change. These criteria do not assure efficacy for behavior change, rather they are assumed to provide the minimum criteria for a program to have the potential for producing behavior change.

### Development of the HBC-I

The original intent of the first of the Tobacco “5 A's,” *Ask*, was to systematically identify all tobacco users and ensure that every patient's tobacco-use status was asked and documented. Since websites inherently assume that a visitor has a specific concern related to the content of the site (eg, the visitor to a smoking cessation site wants to quit smoking or help someone quit smoking), the *Ask* criteria was not included in the HBC-I.

The second strategy, *Advise,* involves practitioners urging tobacco users to quit. For the HBC-I guideline, this was expanded to include advising the individual about a particular behavioral risk and about the need to change the behavior.

*Assess* is the third strategy, in which practitioners assess a patient's willingness to quit. For the variety of behaviors for which programs exist on the Internet, there are many variables, such as self-efficacy and psychosocial variables, which are important for providing appropriate strategies for the individual. Therefore, within the HBC-I, *Assess* was expanded to include the assessment of many possible variables that could impact behavior change.

The Tobacco *Assist* criterion was divided into two separate criteria for the HBC-I. The first, *Assist,* includes providing support, understanding, praise, and reinforcement; describing intervention options; negotiating intervention plans; and/or providing general assistance in making changes. This assistance should include the tailoring of messages based on the assessment from the Internet *Assess* criterion. The second criterion, *Anticipatory Guidance*, was derived from the Tobacco *Assist* strategy and anticipates triggers or challenges that can lead to relapse. The adapted HBC-I *Anticipatory Guidance* criterion includes providing counseling for potential relapse problems and addressing issues of relapse prevention.

*Arrange Follow-up* for Tobacco includes scheduling at least one future contact and suggesting further steps to take during that contact. The HBC-I version includes arranging a follow-up session, reaffirming a plan of action, advising when it would be best to come back to the program, and advising about an appropriate type of follow-up even if the program itself might not provide it.

Two versions of the HBC-I assessment tool were developed to assess these five specific criteria: HBC-I Screener and HBC-I Expanded.

### Application of the HBC-I Screener

The first application of the HBC-I Screener was conducted with 294 websites representing seven targeted behaviors (alcohol use, diet, exercise, smoking, asthma management, depression management, and diabetes management) [3,4]. Sites were identified through online searches, medical information journals, articles, and ads in the popular press. A total of 273 valid websites were evaluated using the HBC-I Screener. Two masters-level reviewers rated the websites on the presence of each of the five HBC-I criteria. The kappa statistic was calculated for each criterion to assess the agreement between the raters, or inter-rater variability. The kappa values for the five categories ranged from 0.84 to 0.93 (mean = 0.88). Kappa values between 0.80 and 1.00 represent almost perfect agreement. A third individual reviewed a site when the two raters disagreed.

Websites were given an overall score ranging from 0 to 5 depending on how many of the criteria were met (Table 1). Overall scores were normally distributed with an average of 1.45 (SD = 1.64) criteria met. Only 8.1% (n = 22) of the websites met all five criteria of the HBC-I Screener, while 7.3% (n = 20) met four. The criterion which most websites met was *Assess* with 51.6% (n = 141). The criterion which the fewest sites met was *Anticipatory Guidance* with 11.4% (n = 31). Table 2 presents the number of websites meeting each of the “5 A's” criteria by behavior. Of those sites meeting four or more of the criteria (n = 42), the behavior most represented was smoking (n = 12; 28.6%), followed by diet (n = 11; 26.2%) [4].

**Table 1:** Number of websites meeting HBC-I screening criteria

**Number of Criteria Met**	**Websites** **No. (%)**
0	113 (41.4)
1	57 (20.9)
2	34 (12.5)
3	27 (9.9)
4	20 (7.3)
5	22 (8.1)

**Table 2:** Number of websites meeting HBC-I screening criteria, by behavior

**Behavior**	**Assess** **No. (%)**	**Advise** **No. (%)**	**Assist** **No. (%)**	**Anticipatory Guidance** **No. (%)**	**Arrange Follow-up** **No. (%)**
Asthma	11 (33)	10 (30)	3 (9)	2 (6)	5 (15)
Alcohol	10 (37)	3 (11)	1 (4)		
Diet	33 (67)	25 (51)	18 (37)	6 (12)	14 (29)
Exercise	30 (73)	19 (46)	15 (37)	7 (17)	14 (34)
Depression	19 (46)	9 (22)	3 (7)	1 (2)	1 (2)
Diabetes	21 (51)	18 (44)	7 (17)	2 (5)	9 (22)
Smoking	16 (44)	24 (67)	14 (39)	13 (36)	10 (28)
**Total websites meeting criteria**	**141 (51.6)**	**108 (39.6)**	**62 (22.7)**	**31 (11.4)**	**54 (19.8)**

Results from a 1-way ANOVA examining the differences in number of criteria met by the different behaviors showed significant results (F_7,272_ = 5.89, *P* < .001, eta^2^ = .14). Websites in the areas of diet, exercise, and smoking met significantly more of the criteria than sites in the areas of alcohol and depression management. Websites in the areas of exercise and smoking received significantly higher overall ratings than sites in asthma management [4].

It is clear from the analyses that the majority of sites readily available to consumers do not meet minimum criteria for health behavior change on the Internet as defined by the HBC-I. The criterion that sites did the best in was *Assess,* and the area with the lowest percentage meeting criterion was *Anticipatory Guidance*. Only 8.1% of the sites received credit in all of the five categories, while 7.3% received four credits. The greatest number of sites meeting four or more of the criteria was in the area of smoking, with diet having the second greatest number. None of the alcohol sites and only one of the depression sites received credit in four or more of the criteria. These results indicate that the development of websites in the areas of diet, exercise, and smoking is much further along in terms of providing the necessary components of health behavior change on the Internet than that in the areas of asthma, alcohol, and depression [4].

### Application of the HBC-I Expanded

An expanded version of the HBC-I Screener was developed to provide more in-depth review criteria concerning the “5 A's” criteria of the HBC-I. Twenty-one behavior change criteria were developed around the five HBC-I screening criteria, and two questions were added to specifically address five major health behavior change theories and variables. The behavioral criteria for the HBC-I Expanded can be found in Cummins et al [1]. As part of the study described above, the HBC-I Expanded was used to evaluate those sites that met a minimum of four of the five HBC-I Screener criteria. Evers et al [3] outlined the results of the reviews, which were conducted by two independent masters-level reviewers on 12 smoking, 11 diet, six exercise, seven diabetes, two asthma, and one depression site. The following highlights are primarily from the “5 A's” criteria:

1. **Advise:** A total of 54% of the sites (n = 20) clearly identified their intended audience, 84% (n = 31) explicitly stated their goals, while 14% (n = 5) implicitly stated their goals. These criteria help guide consumers to appropriate sites.
2. **Assess:** Within each site, assessments were evaluated individually. The types of assessments were dependent on the specific behavior (eg, BMI, exercise level, and stage of change for diet; nicotine dependence, stage of change, and tempting situations for smoking; blood glucose levels for diabetes management).
3. **Assist:** Ninety-seven percent of the sites (n = 36) provided feedback strategies to assist users in achieving health behavior change. The majority of the sites targeted feedback based on the assessments by segmenting the population into specific categories rather than providing individualized feedback. With segmented tailoring, participants were grouped based on a specific variable, and feedback was the same for everyone in that group. However, there is a growing consensus that individually tailored health communication represents one of the most promising modalities for health behavior change [5].
4. **Anticipatory Guidance:** For this criterion, 73% of sites (n = 27) offered some form of anticipatory guidance through information on managing tempting situations (n = 11), preventing relapse to unhealthy behaviors (n = 9), and maintaining the behavior change or staying motivated (n = 17). (Sites could be using more than one type of anticipatory guidance.)
5. **Arrange:** In terms of arranging follow-up, 11% of the sites (n = 4) specified when the user should come back to the program, and 22% (n = 8) used daily email reminders to keep users in touch with the program. Other suggestions ranged from coming back to the site to reassess behavior after a period of time to day-to-day participation.

### Summary

The HBC-I (“5 A's” for Health Behavior Change Treatment on the Internet) criteria were developed to meet the specific needs of behavior change on the Internet. The five basic criteria of the HBC-I (advise, assist, assess, anticipatory guidance, and arrange follow-up) do not assure efficacy for behavior change, rather they outline the minimum criteria for a program to have the potential for providing behavior change. Systematic empirical evaluations of program efficacy would be needed to ultimately demonstrate efficacy. It was discouraging to learn that Evers et al [3] found that none of the sites evaluated included statements about how the program was being evaluated for effectiveness.

Since the development of the two measures of the HBC-I, other studies have used similar frameworks to evaluate behavior change programs on the Internet. For example, Bock et al [6] applied the US Public Health Services “5 A's” [2] to the assessment of the quality of interventions for smoking cessation that are available on the Internet. Two assessment instruments were developed based on the “5 A's” (STS-C and STS-R), in addition to a third which focused on the usability of the website (STS-U). Those instruments were used to evaluate 46 smoking websites. Bock et al [6] found that over 80% of the websites that were evaluated did not include one or more of the key components of tobacco treatment that are recommended in the guidelines [6].

The HBC-I Screener and HBC-I Expanded provide templates for developers of programs, consumers looking for quality sites, and health professionals seeking to recommend the best sites for disease management and prevention. As health behavior change on the Internet matures from the provision of health information to meeting the requirements necessary to produce health behavior change, and as program developers take advantage of the interactive nature of the Internet, criteria such as those in the HBC-I will be essential. Those criteria can instill developers and consumers with confidence that particular programs are at least providing components that meet the minimum conditions for effective behavior change.

## Are They Ready for Us?

In order to maximize the overall impact of health behavior change programs on the Internet, developers and researchers need to move beyond a narrow focus on early adopters and produce a population perspective that includes those not ready, those getting ready, those ready to use such programs, as well as those already participating. This knowledge base can lead directly to the development and dissemination of practical tailored and targeted interventions that can help maximize population participation in Internet programs for health behavior change.

In order to generate both cross-sectional and longitudinal data on a representative population of Internet users' readiness to use the Internet for health behavior change and on the barriers to use, measures were developed based on the Transtheoretical Model of Change [7]. An assessment was administered through two different recruitment methods: proactive recruitment through an invitational phone call to a random sample of Internet users purchased from a list broker, and reactive responses to recruitment letters, posters, or email invitations to participate [8].

### Baseline Assessment

In the first half of 2002, 413 participants completed the first administration of the assessment (baseline). However, only 375 individuals were eligible to participate in the full assessment (eligibility requirements included use of the Internet and specific health risk behaviors). The national sample was similar in demographics to other national samples of Internet users conducted during the same time period. However, the current sample was significantly more highly educated and included more females [8].

The majority of respondents (80.5%) had used the Internet to get health information. However, only 24.7% used the Internet for health behavior change or disease management programs [8]. The majority (62%) had no intention of starting to use health behavior change programs on the Internet in the foreseeable future. Of those who reported using HBC programs on the Internet, 40% were not using programs that met a minimum of four of five criteria on the HBC-I Screener.

### Follow-Up Assessment

The second administration of the survey was conducted one year post baseline. Two hundred and eighty seven participants completed the follow-up survey, resulting in a 77% retention rate. Of those individuals who were using HBC Internet programs at baseline, the majority were no longer using those programs, and 40% had no intention of starting use in the future [8]. The development of measures of the pros and cons of using the Internet for health behavior change and of measures of informed decision making provided insight into the issues surrounding the use or lack of use of such programs [8].

### Summary

The development of a valid, parsimonious set of assessments for readiness to use the Internet for health behavior change (and components related to use, such as informed decision making) provides researchers, program developers, and the health care system with a way to assess their population's readiness to use such Internet programs and thereby guide plans for program development. In addition, the use of such instruments will allow Internet developers, researchers, and program administrators to identify major concerns, benefits, and barriers regarding their populations' use of the Internet-based health behavior change programs.

The results of this survey present a very pessimistic view of the current potential for adoption of the Internet for health behavior change on a population basis. A large majority (about 80%) of the contacted population in the United States was not interested or willing to complete the survey. Of those who were, the clear majority (more than 80%) was not using the Internet for health behavior change and was not intending to. The cons of using the Internet for health behavior change showed no significant decrease as individuals adopted Internet use, indicating that even once individuals start using these programs, the drawbacks of using them are still high. If the Internet is to fulfill its potential as the least costly modality for delivering tailored communication for health behavior change, then considerably more research will be needed to determine what type of interventions, if any, can help significant percentages of populations progress to enhancing their health via the Internet.

The next generation of research needs to take this challenge rather than examining the efficacy of Internet programs with select samples that represent relatively small percentages of at-risk populations. Until the field solves the problem of helping significant percentages of populations progress toward effective action and maintain such action, Internet programs will not be able to realize their potential to be the lowest cost modality for delivering tailored communications that can have the highest impacts on health promotion, disease prevention, and disease management.

## Conclusions

Health behavior change on the Internet appears to be in the early stages of development. A good base has been established, but much work is needed in the future. The examination of the quality and effectiveness of programs available to the general public, as well as descriptive and predictive knowledge about population readiness to participate in such programs, needs further research. Results presented here suggest that many health-related sites do not include the basics of health behavior change, and those that do need improvements in many of the areas believed to be important for the quality and efficacy of health behavior change programs on the Internet. The second portion of the impact equation, participation, also seems to be low, specifically for health behavior change on the Internet. Although many people use the Internet for health in general, few are using health behavior programs, and those that do discontinue use. If the Internet is to fulfill its potential as a cost-effective modality for delivering tailored communication for health behavior change, then considerably more research will be needed to determine both the types of interventions that can help significant percentages of populations progress toward enhancing their health via the Internet and the types of interventions that can help maximize population participation in Internet programs for health behavior change.

## Abbreviations

HBC: health behavior change
